# Supplementary material for: Hungry for Sex: Differential Roles for Ustilago maydis b Locus Components in Haploid Cells vis à vis Nutritional Availability
Source: J Fungi (Basel). 2021 Feb 12;7(2):135. doi: 10.3390/jof7020135 (PMC7918651; doi:10.3390/jof7020135)
Supplement: Supplementary file 1 [file jof-07-00135-s001.pdf]

Journal of *Fungi*

MDPI

Supplementary Files: Hungry for Sex: Differential Roles for *Ustilago maydis b* Locus Components in Haploid Cells vis à vis Nutritional Availability

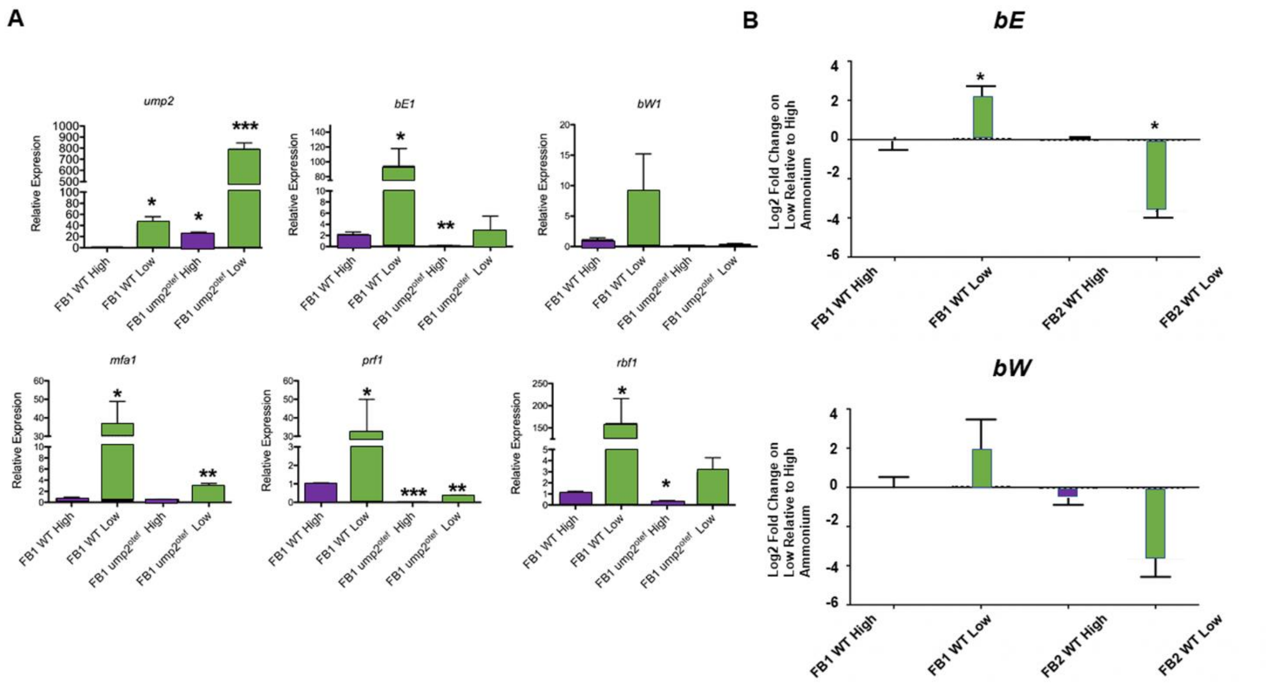

Figure S1. Relative expression of mating-related genes in the FB1 or FB2 background, with and without over-expression of *ump2*. (**A**) the expression of *ump2*, *bE1*, *bW1*, *mfa1*, *prf1*, and *rbf1* as compared at Low- vs. High-NH_4 levels on AM medium. This figure shows the effects of over-expression of *ump2* in this strain background on the responses of these genes to nitrogen-limitation. These qRT-PCR results are adapted from Paul et al. [11], with permission, originally published in *Fungal Biology*, Vol 122, Paul, J.A.; Wallen, R.M.; Zhao, C.; Shi T.; Perlin, M.H. Coordinate regulation of *Ustilago maydis* ammonium transporters and genes involved in mating and pathogenicity, Pages 639–650, Copyright Elsevier (2018). All levels are displayed relative to FB1 WT on High-NH_4 (after normalization to the endogenous housekeeping gene, *eif2B*), using Pfaffl [22]; significance of differences were evaluated comparing each strain/condition relative to wild type FB1 AM-High NH_4 using a student t-test; stars on the graph indicate significance, * $p < 0.05$, ** $p < 0.01$ and *** $p < 0.001$. (**B**), expression of *bE* and *bW* genes in FB1 or FB2 as compared at Low- vs. High-NH_4 levels on AM medium. bE, represents bE1 or bW1, for FB1 and FB2, respectively; bW, represents bE2 or bW2, for FB1 and FB2, respectively. All levels are displayed relative to the respective WT strain on High NH_4 (after normalization to the endogenous housekeeping gene, *eif2B*), as log2 fold changes. Bars represent averages of biological triplicates (for FB1 samples) or duplicates (FB2 samples) and standard errors are indicated in the graphs. The values greater than 0 represent increased expression whereas lower than 0 reflect the decreased expression relative to High-NH_4 conditions. One-way ANOVA followed by Tukey's Multiple Comparison Test was performed in

GraphPad Prism. *, *p*-value <0.05 is considered significant.

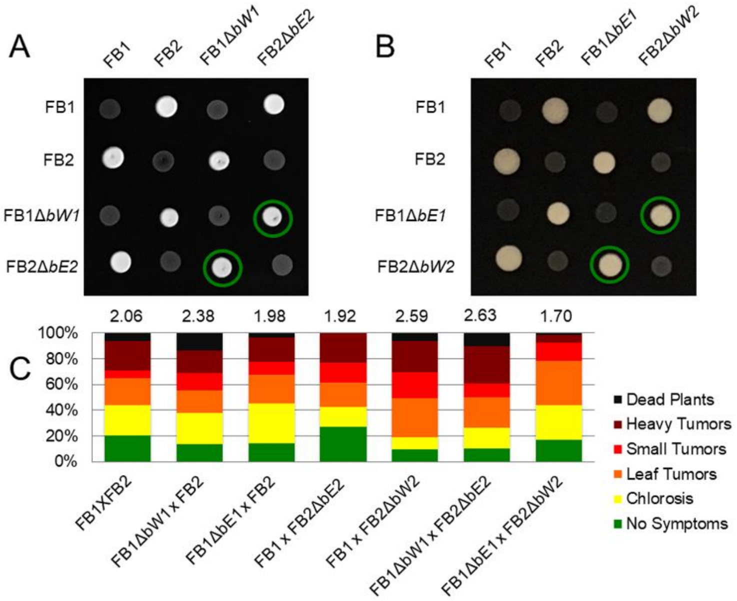

Figure 2. Partial *b* locus deletions have normal responses when mixed with compatible mating partners. Both *bW1* deletions (**A**) and *bE1* deletions (**B**) produced the fuzz positive mating phenotype when plated with compatible mating partners that either contained an intact *b* mating locus, or only contained the allele necessary to form the heterodimeric transcription factor. (**C**) These same strains were able to establish infection to the same degree when injected into maize seedlings with a compatible mating partner.

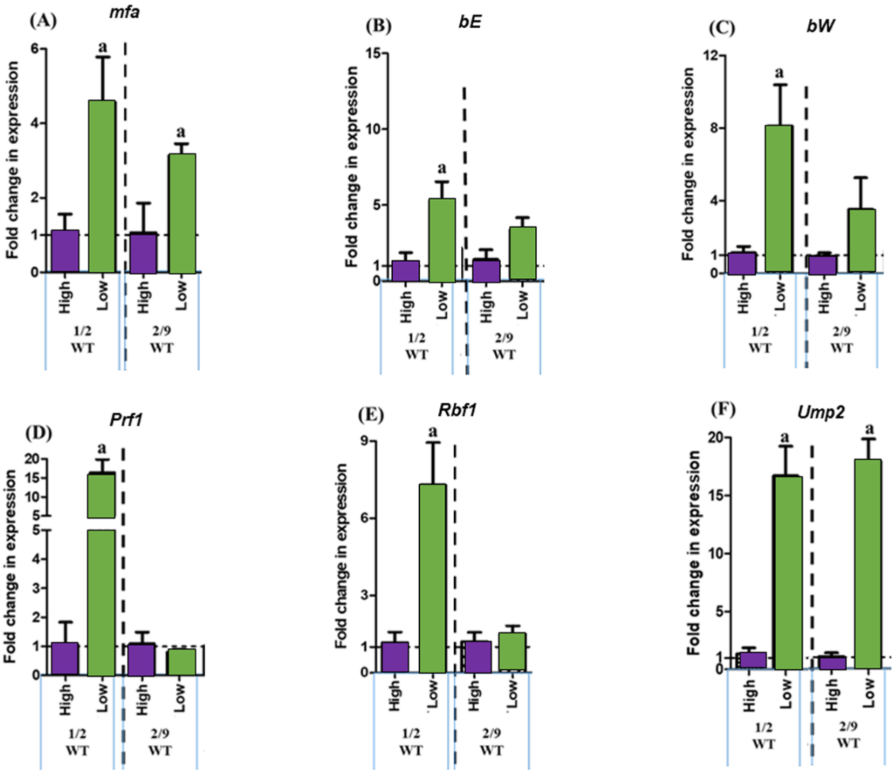

Figure S3. Relative expression of wild type 1/2 and 2/9 (WT) strains on High- and Low-NH_4. In A-F, target genes (*U. maydis* mating pathway genes) are indicated and relative expression levels as normalized transcript levels are shown. High, 30 mM NH_4 medium and Low, 50 μM NH_4 medium. The transcript levels of each target were normalized against *eif2* and are

presented in fold change expression (as calculated from log2-fold changes, using $2^{-\Delta\Delta CT}$) with respect to the same strain grown under High NH_4. Bars represent averages of biological triplicates and standard errors are indicated in the graphs. The values greater than 1 (shown by dotted line) represent increased expression whereas lower than 1 reflect the decreased expression relative to High-NH_4 conditions. One-way ANOVA followed by Tukey's Multiple Comparison Test was performed in GraphPad Prism. p-value <0.05 is considered significant. "a" indicates significant difference in the expression level of target gene for the strain grown on Low NH_4 with respect to same strain grown on High NH_4.

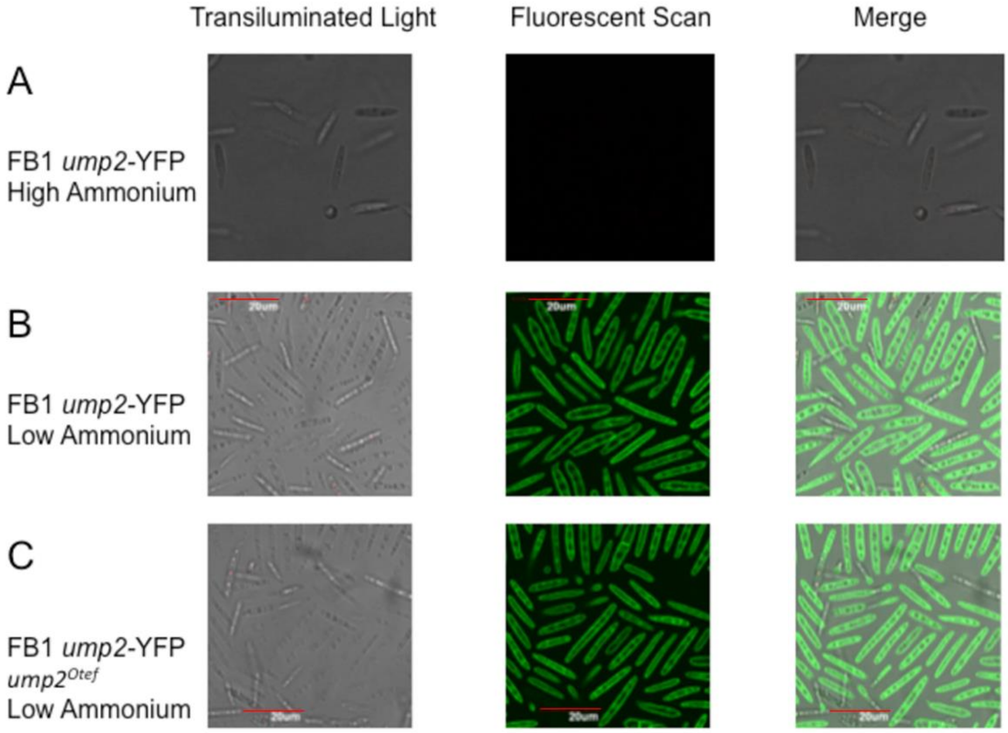

Figure S4. Protein level of *ump2* from native locus as determined by fluorescent microscopy. FB1 *ump2*-YFP (**A**) and (**B**), grown in High and Low NH_4, respectively, and (**C**) FB1 *ump2*-YFP*ump2*^{Potef} grown in High NH_4 (not shown) and Low-NH_4 broth for 24 h and visualized on a confocal microscope. Images are shown for transilluminated light, fluorescent scan, and the merge of the two scans. High, 30 mM NH4 medium, Low, 50 µM NH4. Scale bar, 20 µm. For quantitation of fluorescence intensity, total sums of intensity (integration value) of the entire image area were used to measure YFP intensity, using FV-10 ASW 2.1 software.
